# Supplementary material for: Thalamic deep brain stimulation modulates cycles of seizure risk in epilepsy
Source: Sci Rep. 2021 Dec 20;11:24250. doi: 10.1038/s41598-021-03555-7 (PMC8688461; doi:10.1038/s41598-021-03555-7)
Supplement: Supplementary file 1 — Supplementary Information. [file 41598_2021_3555_MOESM1_ESM.pdf]

Supplementary information

Subject	Sex	Device	Lead location	Dwelling	Record Duration (days)	Seizure count	Gaps
H1	F	RC+S	ANT & Hc	home	338	338	14%
D1	M	NV	frontoparietal neocortex	kennel	394	28	21%
D2	M	NV	frontoparietal neocortex	kennel	395	50	14%
D3	M	NV	frontoparietal neocortex	kennel	170	85	1%
D4	M	NV → RC+S	frontoparietal neocortex	kennel	165	55	17%
D5	M	NV → RC+S	frontoparietal neocortex	kennel	51	15	7%
D6	M	RC+S	ANT & Hc	home	258	45	37%
D7	M	RC+S	ANT & Hc	home	375	11	52%

Supplementary Table 1 Subject characteristics. Gaps indicates the proportion of the recording period without iEEG data. H1 = Human 1. D1 – 7 = Dog 1 – Dog 7. RC+S = Investigational Medtronic Summit RC+S™. NV = NeuroVista Seizure Advisory System. ANT = anterior nucleus of the thalamus. Hc = hippocampus.

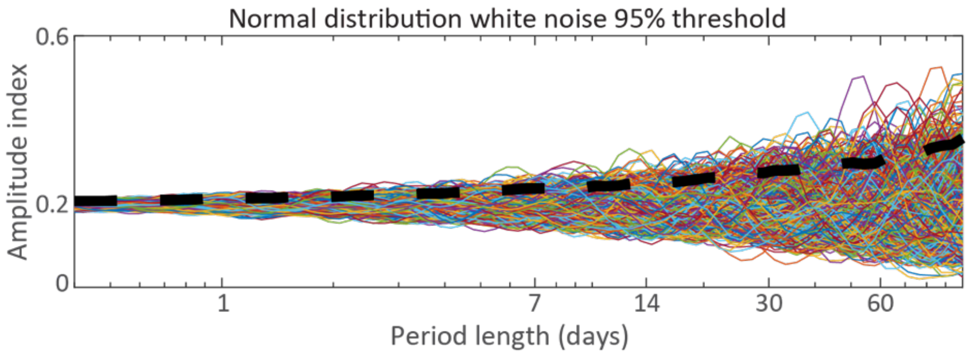

Supplementary Figure 1. Amplitude spectral density of n=1,000 simulations of normally distributed white noise, and 95th percentile line.

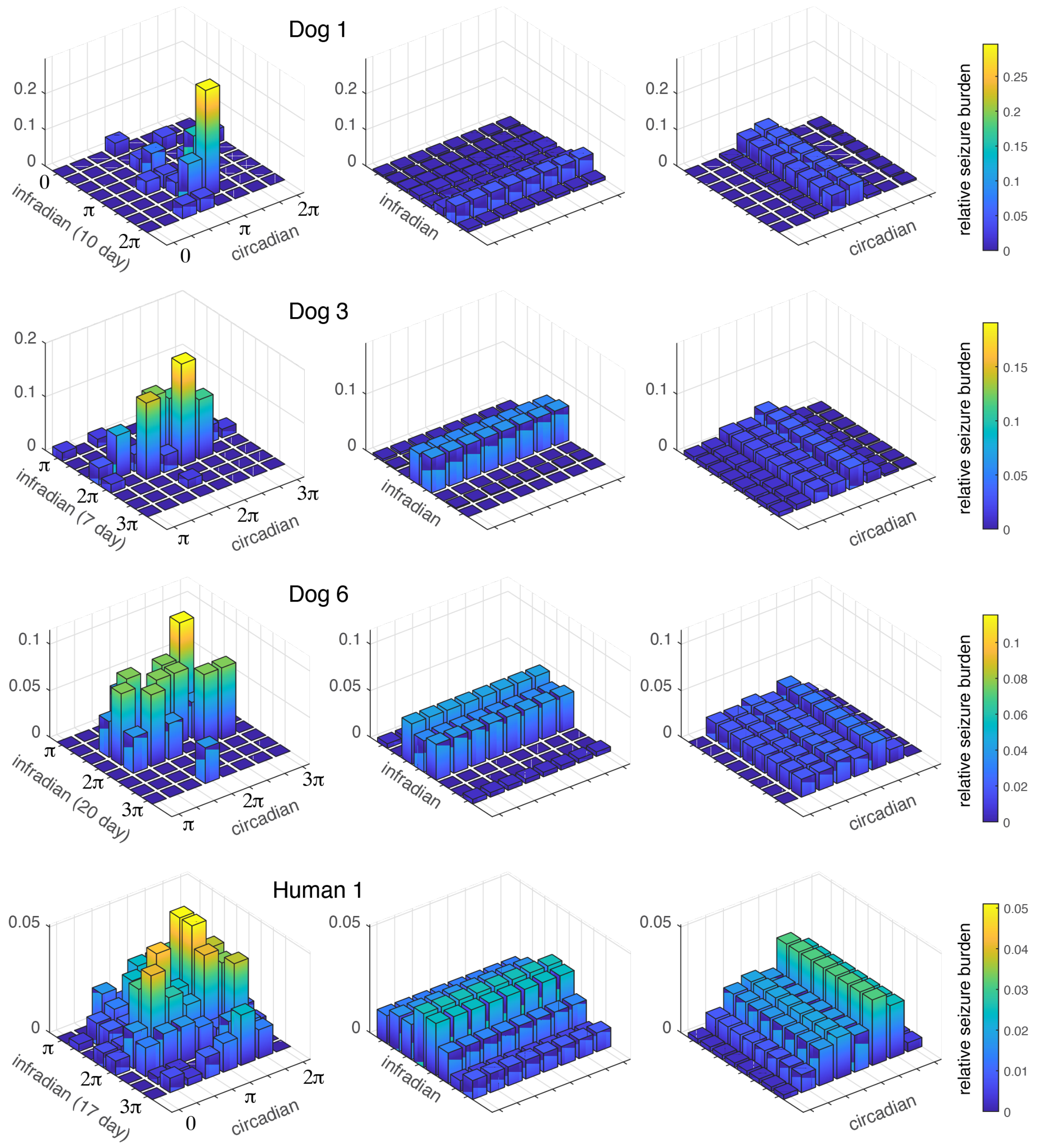

Supplementary Figure 2 Seizure risk phase-phase plots. Seizure risk with phase-phase analysis relative to infradian cycle-only, and circadian cycle-only analysis. Three-dimensional histograms show the burden of seizures in Π/4 x Π/4radian steps, normalized to the total seizure count. For infradian cycle-only and circadian cycle-only plots, seizure counts are constant over the spread of the contrasting (circadian and infradian respectively) cycle.

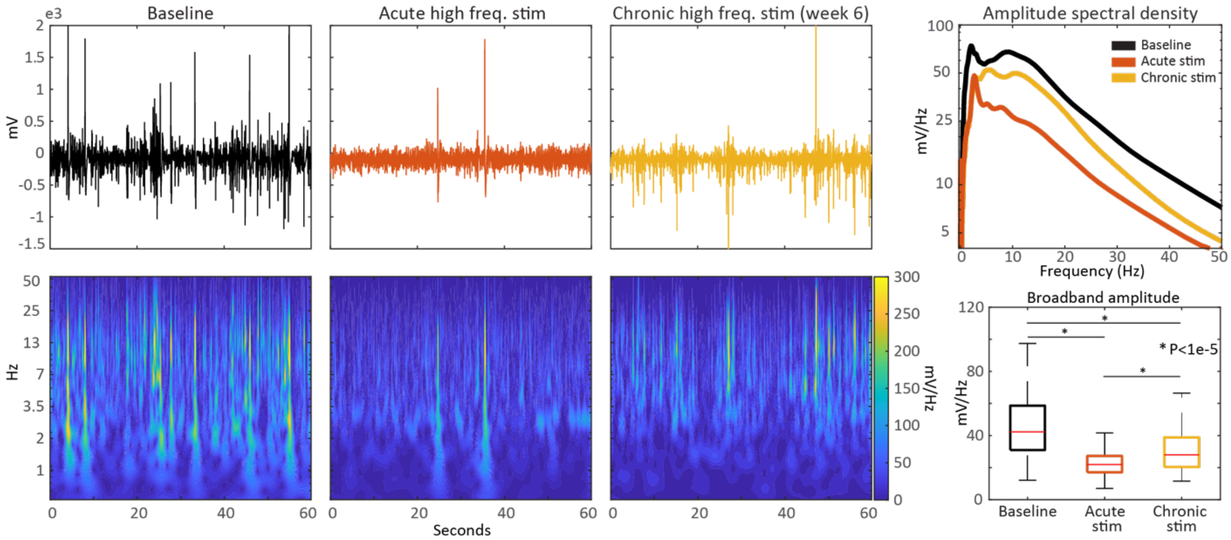

Supplementary Figure 3 Impact of deep brain stimulation on hippocampal local field potential. Representative samples of hippocampal local field potential activity during stimulation off baseline, acute high frequency stimulation, and chronic high frequency stimulation. Broadband amplitude is the 1:50 Hz band mean amplitude.
